# Vascular endothelial growth factor genetic polymorphisms and susceptibility to age-related macular degeneration in Tunisian population

**DOI:** 10.1186/1479-5876-10-S3-P21

**Published:** 2012-11-28

**Authors:** Imen Habibi, Imen Sfar, Fedra Kort, Ahmed Chebil, Rim Bouraoui, Rim Limaiem, Salwa Jendoubi-Ayed, Mouna Makhlouf, Taïeb Ben Abdallah, Khaled Ayed, Leila El Matri, Yousr Gorgi

**Affiliations:** 1 Research Laboratory of Renal Transplantation and Immunopathology (LR03SP01), University Tunis El Manar, Charles Nicolle Hospital, Tunisia; 2 Oculogenetic Research Unit, Dept. B of Ophtalmology, Hedi Rais Institute of Ophthalmology, Tunis, Tunisia

## Introduction

Major genetics factors for age-related macular degeneration (AMD) have recently identified as susceptibility risk factors, underlying the role of the vascular endothelial growth factor (VEGF) system in AMD [1-4].

## Aims

Our purpose was to determine whether (VEGF) gene polymorphisms play a role in either susceptibility risk for age-related macular degeneration (AMD) serum VEGF levels (s-VEGF) variations and treatment with intravitreal bevacizumab in Tunisians.

## Methods

The case-control study included 157 patients with AMD and 207 age-matched controls. In all patients, ophthalmological examinations, visual acuity, optical coherence tomography (OCT), fundus photography and fluorescein angiography were performed. Sixty-two patients were treated with intravitreal bevacizumab. Single nucleotide polymorphism (SNP) genotyping (+936 C>T, +405 C>G and -2578 A>G) were performed using direct sequencing. The serum VEGF was assayed by ELISA (R&D).

## Results

The single SNP +936 TT and +405 CC genotypes were significantly higher in AMD patients than in controls (*p*=0.018 and p<10^-3^, respectively). Haplotype analysis of SNP+936, +405 and -2578 revealed that TGA was associated to exudative form of disease (p<0.0001). However, single allele, genotype and haplotype association analyses showed no significant association with s-VEGF levels variations, clinical forms of AMD or better outcome for distance and reading visual acuity after three bevacizumab injections.

## Conclusions

Our results show that VEGF variants do contribute to the susceptibility to AMD in Tunisian patients. Further expression studies are needed to investigate the potential pharmacologic role of these variants in antiangiogenesis therapy.
